# Is there a decline in speech and swallowing in Amyotrophic Lateral Sclerosis over ten years?

**DOI:** 10.1590/2317-1782/e20240159en

**Published:** 2025-02-10

**Authors:** Regiani Alabarces Mendes, Ivonaldo Leidson Barbosa Lima, Mário Emílio Teixeira Dourado, Maria de Jesus Gonçalves

**Affiliations:** 1 Departamento de Fonoaudiologia, Centro de Ciências da Saúde, Universidade Federal do Rio Grande do Norte – UFRN - Natal (RN), Brasil.; 2 Departamento de Medicina Integrada, Universidade Federal do Rio Grande do Norte – UFRN - Natal (RN), Brasil.

**Keywords:** Motor Neuron Disease, Amyotrophic Lateral Sclerosis, Speech, Dysarthria, Deglutition, Swallowing Disorder

## Abstract

**Purpose:**

To analyze the evolution of speech and swallowing decline in patients with amyotrophic lateral sclerosis (ALS) over a ten-year period.

**Methods:**

A retrospective and longitudinal cohort study. Data were collected using the Amyotrophic Lateral Sclerosis Functional Rating Scale-Revised (ALSFRS-R) from 101 medical records of ALS patients treated at the multidisciplinary neuromuscular diseases clinic of a University Hospital over a ten-year period. The data were statistically analyzed, adopting a significance level of p<0.05.

**Results:**

The analysis of the studied functions indicated that speech, swallowing, and salivation are altered over ten years in ALS. There are differences in patterns between the variables sex and disease type concerning symptoms related to dysarthria and dysphagia in these individuals, which may indicate the rate of progression over a given time interval.

**Conclusion:**

There is a decline in speech and swallowing over ten years in ALS. The bulbar type leads to a faster decline in the studied functions than the spinal type.

## INTRODUCTION

Amyotrophic lateral sclerosis (ALS) is a progressive neurodegenerative disease of unknown etiology, which affects motor neurons through the combination of several associated factors, and can be either genetically or sporadically derived. It affects individuals of both sexes, typically after the age of 50^(1)^.

As a disease characterized by the degeneration of motor neurons located both in the cerebral cortex, brainstem (NMS), and spinal cord (NMI)^(1)^, impairment of speech and swallowing functions is irreversible, resulting in significant loss of quality of life for these individuals^(2)^. Patients affected by this condition frequently present disorders related to deficits in swallowing, breathing, and speech processes, among other comorbidities^(3)^.

There are currently no established standards for early detection or monitoring the progression of dysarthria and dysphagia symptoms, despite these being considered severe conditions that significantly compromise these vital functions in ALS patients.

Accurate markers of motor decline are needed in clinical care to reduce diagnostic uncertainty and minimize the progression of these symptoms during the disease's course^(4,5)^. Few studies have critically evaluated the degenerative progression of neurovegetative functions in patients with neuromuscular diseases, particularly in individuals with ALS^(6,7)^. When such studies are conducted in heterogeneous samples with various associated pathologies, the research outcomes are often compromised^(8)^.

This highlights the need for research aimed at systematically and early detecting the degenerative progression of these functions in patients with similar samples, using either objective or subjective mechanisms. There are reports of practices for assessing the progression of these disorders based on subjective clinical judgments and some objective studies of specific symptoms such as sialorrhea and levels of dysphagia. However, studies that evaluate the progression of speech and swallowing symptoms in association with variables such as sex or disease type remain scarce in the literature^(7-9)^.

The implementation of monitoring of dysarthria and dysphagia would support speech-language pathology interventions, enhancing the processes of evaluation, diagnosis, counseling, and rehabilitation in ALS^(10-12)^. In this regard, the aim of this study was to analyze the evolution of speech and swallowing decline in patients with amyotrophic lateral sclerosis (ALS) over a ten-year period.

## METHOD

This study is a retrospective, longitudinal, cohort, and quantitative research, approved by the Research Ethics Committee (CEP) under opinion no. 3.548.214, with exemption from the Informed Consent Form (ICF).

Data collection was conducted using the Amyotrophic Lateral Sclerosis Functional Rating Scale-Revised (ALSFRS-R)^(13)^, an instrument used to assess the levels of functional changes, closely aligned with objective measures of muscle strength and pulmonary function used in clinical trials. This scale is specifically designed to evaluate ALS patients and aims to sensitively measure the assessed items, reproducing the clinical course of these patients in terms of motor functions, functional abilities, levels of self-sufficiency, and areas related to feeding, ambulation, self-care, and communication^(4)^. The instrument can be administered by healthcare professionals, such as speech-language pathologists, with experience in caring for ALS patients and the expertise to assess neuromuscular function and monitor disease progression.

The ALSFRS-R consists of twelve items for evaluation: 1 - Speech, 2 - Salivation, 3 - Swallowing, 4 - Writing, 5 - Handling food and utensils (for patients without gastrostomy), 6 - Dressing and hygiene, 7 - Turning in bed and adjusting bed sheets, 8 - Walking, 9 - Climbing stairs, 10 - Dyspnea, 11 - Orthopnea, and 12 - Respiratory failure^(13)^. For this study, only the items “speech,” “salivation,” and “swallowing” were considered. Each item has a score from 0 to 4, with a maximum score of 40 points, where 4 represents normal function and 0 represents the worst score.

The inclusion criteria for the medical records were: adult patients with a clinically defined diagnosis of ALS, of both sexes, treated at the neuromuscular diseases clinic of a University Hospital in the city of Natal, Rio Grande do Norte, over a 10-year period, who had a record of ALSFRS-R evaluation in their medical records.

The exclusion criteria were: patients with a diagnosis of ALS associated with other pathologies, incomplete or missing data in the medical records, and errors or inconsistencies in filling out the scale, such as erasures.

Initially, 173 medical records from the clinic were reviewed, and 101 were selected for this study after applying the eligibility criteria. Thus, the studied variables were obtained from the data collection of 101 medical records, comprising 64 men and 37 women, with an average age of 56 years (±20.5). Regarding ALS type, 27 subjects had bulbar ALS and 74 had spinal ALS.

The data were analyzed using the Kaplan-Meier method, Log-rank test, and Cox regression test^(14,15)^. The Kaplan-Meier method estimated the probability that survival functions would remain stable or deteriorate over time^(14)^.

In this study, the percentage of survival function maintenance over time was considered, or in other words, the percentage of function maintenance over a given time interval (t), considering the first and last record on the scale to determine the event outcome (worst score = 0). Thus, the progression of changes in speech, salivation, and swallowing functions was analyzed by using the first and last scores for each individual, with ALSFRS-R records from their physical medical records.

The Log-rank test was used to compare Kaplan-Meier curves between two groups and to determine if there was a statistical difference between the curves^(15)^. The grouping for this comparison of curves was based on disease type – bulbar ALS and spinal ALS – and on sex (female and male). A significance level of 5% was used for all analyses.

The Cox regression test was also used to determine the risks associated with the evolution of functional changes in the evaluated items, in comparison to other variables, such as disease type and sex.

## RESULTS

For the analysis of the functions using the Kaplan-Meier method, all observations of the assessed functions were considered, from the first to the last record on the functional scale, over a 10-year period.

**Figure 1:**
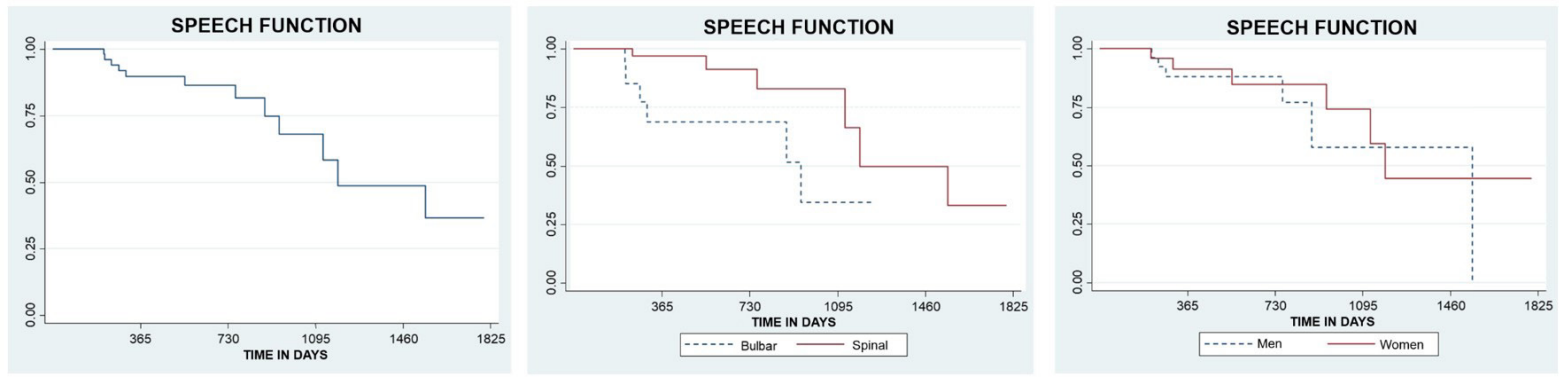
Kaplan-Meier curves estimating the maintenance of speech function over time in patients with ALS, comparing the maintenance estimates according to ALS type and participant sex

Regarding speech, the median estimate for maintaining this function was 2,648 days (7 years; 3 months; 3 days), with a standard deviation of 0.119 and a 95% confidence interval across the individuals analyzed, considering the total group. It was observed that there is a 50% probability of maintaining speech function for approximately 1,248 days (3 years; 5 months; 3 days) in the individuals analyzed (Figure 1).

However, when comparing individuals with bulbar ALS and those with spinal ALS, it was found that the estimate for individuals with bulbar ALS to maintain this function was around 883 days (2 years; 5 months; 3 days), while individuals with spinal ALS reached the same outcome in approximately 2,648 days (7 years; 3 months; 3 days). Individuals with bulbar ALS maintained 68% of this function between 304 and 853 days (10 months; 4 days to 2 years; 4 months; 3 days), while individuals with spinal ALS managed to maintain 66% of the function between approximately 1,553 and 1,796 days (4 years; 3 months; 3 days to 4 years; 11 months; 6 days). The standard deviation was 0.17 for bulbar ALS and 0.13 for spinal ALS (Figure 1).

In the Log-rank test, it was found that there was a significant difference between individuals with bulbar ALS and spinal ALS in the progression of the disease, regarding the maintenance of speech, with a p-value of 0.0027. Cox regression analysis revealed that the risk of individuals with bulbar ALS losing speech before individuals with spinal ALS was 5.34 times greater.

Regarding sex, males had a speech maintenance interval between 1,553 and 2,800 days (4 years; 3 months; 3 days and 7 years; 8 months; 5 days), while females had an interval between 1,126 and 1,796 days (3 years; 2 months; 11 days and 4 years; 11 months; 6 days). It was observed that males maintained 73% of speech function between 883 and 1,248 days (2 years; 5 months; 3 days and 3 years; 5 months; 3 days), while females maintained 77% of this function between 943 and 1,065 days (2 years; 7 months; 3 days and 2 years; 11 months; 5 days) (Figure 1).

Regarding the loss of speech function, the Log-rank test showed a p-value of 0.567, indicating that there was no statistical difference in the progression of speech loss between the two sexes. Cox regression comparison determined that the risk of females losing speech function before males was 1.35 times greater.

**Figure 2:**
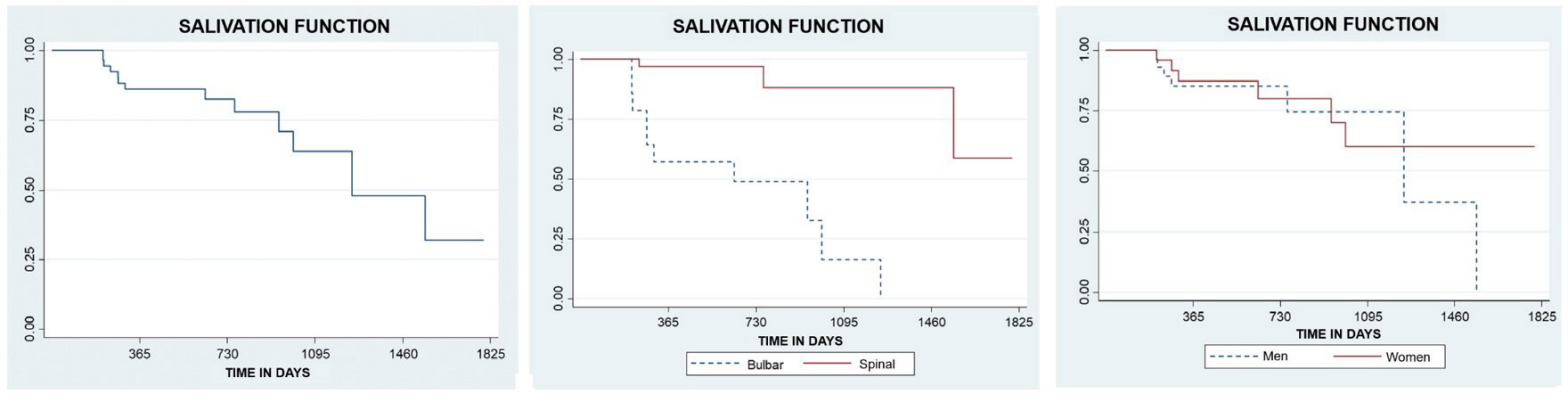
Kaplan-Meier curves estimating the maintenance of salivation function over time in patients with ALS, comparing the maintenance estimates according to ALS type and participant sex

In the analysis of salivation function, considering the increase in saliva accumulation in the oral cavity, it was observed that individuals with ALS maintained good salivation functionality between 1,553 and 1,796 days (4 years; 3 months; 3 days and 4 years; 11 months; 6 days). At an estimated time of 1,248 days (3 years; 5 months; 3 days), these subjects still maintained 65% of the salivation function, with a standard deviation of 0.12 and a 95% confidence interval (Figure 2).

In Figure 2, the difference in the maintenance of salivation function between individuals with bulbar ALS and spinal ALS can be observed. It was found that individuals with spinal ALS maintained this function for a longer period than those with bulbar ALS. While individuals with bulbar ALS maintained 57% of this function between 304 and 518 days (10 months; 4 days to 1 year; 5 months; 3 days), those with spinal ALS maintained 82% of this function between 1,553 and 1,796 days (4 years; 3 months; 2 days to 4 years; 11 months; 9 days).

The Log-rank test revealed a statistical difference between the two types, with a p-value of < 0.0001. Cox regression showed that patients with bulbar ALS had a 21.45 times higher risk of developing sialorrhea complaints compared to patients with spinal ALS.

When comparing males and females, the curve showed that males maintained 80% of salivation function between 761 and 1,064 days (2 years; 1 month; 1 day and 2 years; 11 months; 4 days), while females maintained 81% of this function between 638 and 850 days (1 year; 9 months; 1 day to 2 years; 4 months) (Figure 2).

When these variables were analyzed by the Log-rank test, no statistical difference was observed in salivation function between sexes (p = 0.683). In Cox regression, females showed a 1.24 times higher risk of progressing to a worsening of this complaint compared to males.

**Figure 3:**
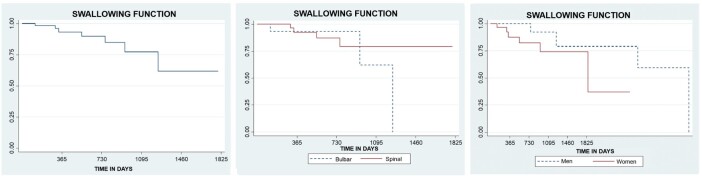
Kaplan-Meier curves estimating the maintenance of swallowing function over time in patients with ALS, comparing the maintenance estimates according to ALS type and participant sex

In the statistical analysis of swallowing function, using the Kaplan-Meier method, the median was between 0.65 and 0.48, indicating that for the outcome of this event in individuals with ALS, it takes between 2,640 and 2,800 days (7 years; 2 months; 25 days to 7 years; 8 months; 5 days), with a standard error of 0.12. In Figure 3, it can be observed that 82% of this function is maintained between 943 and 1,188 days (2 years; 7 months; 3 days and 3 years; 3 months; 3 days).

When this function was compared between individuals with bulbar ALS and spinal ALS, it was found that 93% of the swallowing function is preserved between 120 and 883 days (4 months and 2 years; 5 months; 3 days) in patients with bulbar ALS. In patients with spinal ALS, 90% of this function is maintained between 546 and 730 days (1 year; 6 months; 3 days and 2 years) (Figure 3).

In patients with spinal ALS, swallowing function was maintained between 2,800 and 3,259 days (7 years; 8 months; 5 days and 8 years; 11 months; 3 days). The Log-rank test demonstrated no statistical difference when comparing individuals with bulbar ALS and spinal ALS, with a p-value of 0.107.

In Cox regression, the result indicated that individuals with bulbar ALS have a 3.24 times higher risk of losing swallowing function compared to individuals with spinal ALS.

When the changes in this function were compared between sexes, the Kaplan-Meier test showed that 92% of swallowing function was maintained in males between 761 and 1,064 days (2 years; 1 month; 1 day and 2 years; 11 months; 4 days). In females, 74% of this function was maintained between 943 and 1,796 days (2 years; 7 months; 3 days and 4 years; 11 months; 6 days) (Figure 3).

The Log-rank test did not show a difference in this comparison, with a p-value of 0.081. Regarding this variable, Cox regression indicated that females have a 3.82 times higher risk of reaching function decline more rapidly.

## DISCUSSION

The results obtained regarding speech are in agreement with the literature, as changes in this function become more evident with disease progression^(16,17)^. Patients with ALS, regardless of subtype, progress to dysarthria and often to loss of speech. Dysarthria is characterized by impairment of the motor components of speech (articulation, resonance, phonation, prosody, and respiration), typically associated with bulbar or pyramidal tract involvement, affecting speed, amplitude, firmness, and rendering speech imprecise and unintelligible, which may progress to anarthria^(18,19)^.

When considering the ALS subtype, bulbar or spinal, the analysis showed that individuals with bulbar ALS retain a percentage of this function for a shorter period than those with spinal ALS. One study indicated that only 30% of individuals with ALS begin with bulbar onset^(20)^, which corroborates the results of our study, considering the sample size, which shows a higher percentage of individuals with spinal ALS. However, bulbar-type subjects experience speech loss over a short period^(4)^, as bulbar ALS involves the upper motor neurons (UMNs), lower motor neurons (LMNs), or both, located in the cortex and brainstem, causing difficulties in verbal fluency and voice, making speech slower, breathy, and hypernasal^(1,5,10)^.

However, these studies do not mention the timeframe in which these changes occur. The survival analysis using the Kaplan-Meier method, applied in our study, allowed us to quantify the estimated preservation of speech function, and consequently, to make informed decisions regarding the timing of therapeutic interventions and the strategies to be adopted in this process. This analysis highlights the importance of using time markers in the clinical and research settings of neuromuscular diseases.

The time marker has emerged as a crucial tool in the evaluation and monitoring of neuromuscular diseases, complementing traditional measures of muscle function and quality of life^(21-23)^. The importance of this marker lies in its ability to capture subtle and progressive changes over time, providing data on disease progression and the effectiveness of therapeutic interventions^(21-23)^.

Previous studies^(24-26)^ have demonstrated that the rate of progression in neuromuscular diseases can vary widely between individuals and across different disease types, such as Duchenne muscular dystrophy, ALS, and spinal muscular atrophy. The use of time markers allows for a more personalized approach, identifying patterns of progression that may not be evident through sporadic clinical assessments^(24-26)^.

In ALS^(21,25)^, longitudinal assessment of functional and clinical-temporal changes in patients can provide critical information about disease progression and the understanding of its clinical variability, as well as facilitate the development of personalized therapeutic approaches and the analysis of intervention effectiveness.

The Cox regression analysis revealed that the risk of bulbar-type individuals losing speech before those with spinal ALS is 5.34 times higher, supporting the findings of previous studies on these comparisons^(4,10)^.

Regarding salivation analysis, the result considering all subjects allowed for the identification of the time interval during which this function is preserved. Difficulty in expelling saliva from the oral cavity is known as sialorrhea, which can lead to stasis and progress to the risk of aspiration pneumonia, a consequence of weakness and incoordination of the muscles involved in swallowing function due to dysphagia^(27,28)^.

The application of botulinum toxin is commonly used in the treatment of sialorrhea in patients with ALS, as this dysfunction is present in 50% of these patients, suggesting an alteration in the mechanism involved in saliva swallowing^(28)^. The use of botulinum toxin improves the quality of life for these patients. Observing progression, as described in the analysis of this study, can help determine the most appropriate time for the use of this therapy.

In this study, the progression of swallowing dysfunction occurred in a gradual manner, being faster in individuals with bulbar ALS compared to those with spinal ALS. Swallowing is a complex function that involves the neuromuscular action of structures such as the cortex, cerebellum, and brainstem, which are closely related to oropharyngeal events^(4,5,8,12)^.

Indeed, in individuals with bulbar ALS, these structures are primarily affected, resulting in degeneration that quickly impairs swallowing function. Dysphagia is common at all stages of swallowing in these individuals, even in the absence of patient complaints, as observed in evaluations of the oral preparatory, oral, and pharyngeal phases of swallowing, using videoendoscopy in people with ALS^(29)^.

Regarding the reduced preservation of swallowing function in bulbar ALS compared to spinal ALS, other studies have objectively assessed oropharyngeal manifestations in ALS, comparing the spinal and bulbar forms of the disease. These studies found that oropharyngeal manifestations occur more frequently in patients with bulbar ALS than in those with the spinal form^(4,8,16-18)^.

Concerning the gender variable, all analyses reviewed indicated faster progression in women with respect to swallowing, speech, and salivation. Although other studies do not compare the functional alterations studied in relation to this variable, a prevalence study on ALS in the city of Porto Alegre, Brazil, demonstrated that men are more commonly affected by this disease than women^(30)^.

It is worth noting that the analysis in this study allowed us to observe and quantify, in days, the maintenance of speech, salivation, and swallowing. These data are important for clinical speech-language pathology practice with individuals with ALS, as they support: the process of assessing symptoms presented by patients; prognosis; intervention planning and evaluation of their effectiveness; monitoring disease progression; and communication with the patient and their family.

As study limitations, we highlight the exclusion of medical records due to, primarily, the observation of errors or inconsistencies in the completion of the scale or other registration data. These issues emphasize the importance of proper documentation of data and the need for training health professionals in the effective application of functional scales. Future studies are recommended to analyze other aspects of the scale and compare its data with those from longitudinal, clinical, and objective assessments of different ALS subtypes.

## CONCLUSION

There is a progressive decline in speech, salivation, and swallowing in patients with ALS over ten years. Individuals with spinal ALS maintain these functions for a longer period than those with bulbar ALS. Additionally, women with ALS exhibit a faster decline in the analyzed functions compared to men with ALS.
