# Supplementary material for: Is there a decline in speech and swallowing in Amyotrophic Lateral Sclerosis over ten years?
Source: Codas. 2025 Feb 10;37(2):e20240159. doi: 10.1590/2317-1782/e20240159en (PMC11813176; doi:10.1590/2317-1782/e20240159en)
Supplement: Translation

# Translation: Há declínio da fala e da deglutição na Esclerose Lateral Amiotrófica ao longo de dez anos?

**DOI:** 10.1590/2317-1782/e20240159pt

**Authors:** Regiani Alabarces Mendes, Ivonaldo Leidson Barbosa Lima, Mário Emílio Teixeira Dourado, Maria de Jesus Gonçalves

**Affiliations:** 1 Departamento de Fonoaudiologia, Centro de Ciências da Saúde, Universidade Federal do Rio Grande do Norte – UFRN - Natal (RN), Brasil.; 2 Departamento de Medicina Integrada, Universidade Federal do Rio Grande do Norte – UFRN - Natal (RN), Brasil.

**Keywords:** Doença dos Neurônios Motores, Esclerose Amiotrófica Lateral, Fala, Disartria, Deglutição, Transtornos de Deglutição

## Abstract

**Objetivo:**

Analisar a evolução do declínio da fala e da deglutição em pacientes com esclerose lateral amiotrófica (ELA) ao longo de dez anos.

**Método:**

Estudo de coorte, retrospectivo e longitudinal. Foi realizado por meio da coleta de dados da escala funcional Amyotrophic Lateral Sclerosis - Functional Rating Scale-Revised (ALSFRS-R) em 101 prontuários de pacientes com ELA atendidos no ambulatório multidisciplinar de doenças neuromusculares de um Hospital Universitário, em um período de dez anos. Os dados foram analisados estatisticamente, adotando o valor de p<0,05.

**Resultados:**

A análise das funções estudadas indicou que a fala, a deglutição e a salivação são alteradas ao longo de dez anos na ELA. Há diferenças de padrões entre as variáveis sexo e tipo da doença, nos sintomas relacionados à disartria e à disfagia nesses indivíduos, podendo precisar em dias o quanto esta evolução ocorre ao longo de um determinado intervalo de tempo.

**Conclusão:**

Há declínio da fala e da deglutição ao longo de dez anos na ELA. O tipo bulbar provoca um declínio mais rápido das funções estudadas, do que o tipo espinhal.

## INTRODUÇÃO

A esclerose lateral amiotrófica (ELA) é uma doença neurodegenerativa progressiva, de etiologia desconhecida, que atinge os neurônios motores por meio da combinação de vários fatores associados, podendo ser de origem genética ou esporádica. Afeta pessoas de ambos os sexos, normalmente após os 50 anos de idade^(1)^.

Por ser uma doença caracterizada pela degeneração dos neurônios motores, localizados tanto no córtex cerebral, no tronco encefálico (NMS) quanto na medula espinhal (NMI)^(1)^, o comprometimento das funções da fala e deglutição é irreversível, trazendo inúmeros prejuízos à qualidade de vida desses indivíduos^(2)^. Pacientes acometidos por esta patologia, frequentemente, apresentam distúrbios relacionados a déficits nos processos de deglutição, respiração e fala, dentre outras comorbidades^(3)^.

Ainda não existem padrões estabelecidos para detecção precoce ou monitoramento da progressão dos sintomas da disartria e da disfagia, mesmo sendo considerados graves e com comprometimento significativamente preocupante dessas funções vitais em pacientes com ELA.

Marcadores precisos de declínio motor são necessários nos cuidados clínicos para reduzir a incerteza diagnóstica e minimizar a progressão desses sintomas durante a evolução da doença^(4,5)^. Observa-se que há poucas pesquisas que avaliem criteriosamente a evolução degenerativa das funções neurovegetativas em pacientes com doenças neuromusculares, principalmente em indivíduos com ELA^(6,7)^. Quando estes estudos são realizados em amostras heterogêneas, com diversas patologias associadas, os resultados da pesquisa ficam comprometidos^(8)^.

Isso evidencia a carência em pesquisas que visem verificar precocemente e sistematicamente a progressão degenerativa dessas funções em pacientes com amostras semelhantes, através da utilização de mecanismos objetivos ou subjetivos. Há relatos de práticas para avaliação da progressão desses distúrbios com base em julgamentos clínicos subjetivos e alguns estudos objetivos de sintomas específicos como sialorreia e níveis de disfagia, porém continuam escassos na literatura estudos que avaliem a progressão de sintomas na fala e na deglutição associadas a variáveis como sexo ou tipo da doença^(7-9)^.

A implementação do monitoramento de disartria e disfagia auxiliará a atuação fonoaudiológica, favorecendo o processo de avaliação, diagnóstico, orientação e reabilitação na ELA^(10-12)^. Nesse sentido, o objetivo deste trabalho foi analisar a evolução do declínio da fala e da deglutição em pacientes com esclerose lateral amiotrófica (ELA) ao longo de dez anos.

## MÉTODO

A presente pesquisa é um estudo de coorte, retrospectivo, longitudinal e quantitativo, aprovado pelo Comitê de Ética em Pesquisa (CEP) pelo parecer nº 3.548.214, com dispensa do Termo de Consentimento Livre e Esclarecido (TCLE).

Foi utilizada para coleta de dados a escala funcional *Amyotrophic Lateral Sclerosis - Functional Rating Scale - Revised* (ALSFRS-R)^(13)^, um instrumento utilizado para avaliar os níveis de alterações funcionais, estando em estreita concordância com medidas objetivas de força muscular e função pulmonar utilizadas em testes clínicos. Esta escala é específica para avaliar pacientes com ELA e tem por objetivo medir sensivelmente os itens avaliados, reproduzindo o curso clínico desses pacientes, nas funções motoras e habilidades funcionais, nos níveis de autossuficiência, nas áreas relativas à alimentação, deambulação, autocuidado e comunicação^(4)^. O instrumento pode ser aplicado por profissionais de saúde, como o fonoaudiólogo, que tenham experiência no cuidado de pacientes com ELA e aptidão para avaliar a função neuromuscular e acompanhar a progressão da doença.

A ALSFRS-R apresenta doze itens para avaliação: 1- fala, 2-salivação, 3-deglutição, 4-escrita, 5-manipulação de alimentos e utensílios (pacientes sem gastrostomia), 6-vestuário e higiene, 7- virar na cama e ajustar a roupa de cama, 8-andar, 9-subir escadas, 10-dispnéia, 11-ortopnéia e 12-insuficiência respiratória^(13)^. Para esta pesquisa, foram considerados apenas os itens “fala”, “salivação” e “deglutição”. Cada um dos itens tem uma pontuação de 0 a 4, com escore máximo de 40 pontos, em que 4 representa o padrão de normalidade e 0 representa o pior escore.

Os critérios de inclusão dos prontuários foram: pacientes adultos com diagnóstico clínico definido de ELA, de ambos os sexos, atendidos no ambulatório de doenças neuromusculares de um Hospital Universitário da cidade de Natal, Rio Grande do Norte, em um período de 10 anos, que tiveram registro de avaliação da escala funcional ALSFRS-R no prontuário.

Os critérios de exclusão foram: pacientes com diagnóstico de ELA associada a outras patologias, observação de dados incompletos ou ausentes no prontuário, e identificação de erros ou inconsistências no preenchimento da escala, como rasura no preenchimento.

Inicialmente, investigaram-se os dados de 173 prontuários do ambulatório e 101 foram selecionados para este estudo, após análise dos critérios de elegibilidade. Desse modo, as variáveis estudadas foram obtidas por meio do levantamento de dados em 101 prontuários, sendo 64 homens e37 mulheres, com uma média de idade de 56 anos (±20,5). Em relação ao tipo da ELA, 27 sujeitos apresentavam a ELA do tipo bulbar e 74 a ELA do tipo espinhal.

Os dados foram analisados pelo método Kaplan-Meier, teste de Log-rank e teste de Regressão de Cox^(14,15)^. O método Kaplan-Meier estimou a probabilidade das funções de sobrevida permanecerem ou se deteriorarem ao longo do tempo^(14)^.

No presente estudo, considerou-se o percentual da manutenção da função de sobrevida ao longo do tempo ou, em outras palavras, qual o percentual de manutenção da função permanece em um determinado intervalo de tempo (t), considerando o primeiro e o último registro na escala para se chegar ao desfecho do evento (pior escore=0). Assim, pode ser analisada a progressão das alterações das funções da fala, salivação e deglutição, utilizando a primeira e a última pontuação de cada indivíduo, com registro da ALSFRS-R no prontuário físico.

O teste de Log-rank foi utilizado para comparar as curvas de Kaplan-Meier de dois grupos e determinar se houve diferença estatística entre as curvas^(15)^. A divisão em grupos para essa comparação das curvas foi realizada com base no tipo da doença – ELA do tipo bulbar e ELA do tipo espinhal – e no sexo, feminino e masculino. O nível de significância utilizado em todas as análises foi de 5%.

Também foi utilizado o teste de regressão de Cox para determinar os riscos entre a evolução das alterações das funções dos itens avaliados em comparação a outras variáveis, tais como: tipo da doença e sexo.

## RESULTADOS

Para análise das funções pelo método Kaplan-Meier, considerou-se todas as observações das funções analisadas, desde o primeiro até o último registro da escala funcional, em um intervalo de tempo de 10 anos.

Em relação à fala, a estimativa mediana para a manutenção desta função foi de 2.648 dias (7a;3m;3d), com desvio-padrão de 0,119 e um intervalo de confiança de 95% entre os indivíduos analisados, considerando o grupo total. Verificou-se que existe uma probabilidade de manter 50% da função da fala em aproximadamente 1.248 dias (3a;5m;3d) para os indivíduos analisados (Figura 1).

**Figura 1:** Curvas de Kaplan-Meier com estimativa de manutenção da função da fala ao longo do tempo em pacientes com ELA e comparando a estimativa de manutenção de acordo com o tipo de ELA e em função do sexo dos participantes

Entretanto, ao comparar indivíduos com ELA bulbar e indivíduos com ELA espinhal, observou-se que a estimativa para indivíduos com ELA do tipo bulbar manterem esta função fica em torno de 883 dias (2a;5m;3d), enquanto os indivíduos com ELA do tipo espinhal alcançam esse mesmo desfecho em aproximadamente 2.648 dias (7a;3m;3d). Indivíduos com ELA do tipo bulbar mantêm 68% dessa função entre 304 e 853 dias (10m;4d e 2a;4m;3d), enquanto os indivíduos com ELA do tipo espinhal conseguem manter 66% da função no intervalo de aproximadamente 1.553 a 1.796 dias (4a;3m;3d a 4a;11m;6d). O desvio padrão foi de 0,17 no tipo bulbar e 0,13 no tipo espinhal (Figura 1).

No teste de Log-rank, verificou-se que há diferença significativa entre indivíduos com ELA do tipo bulbar e ELA do tipo espinhal, durante o tempo de progressão da doença, em relação à manutenção da fala, com valor de p=0,0027. A análise pelo teste de regressão de Cox revelou que o risco de indivíduos com ELA do tipo bulbar perderem a fala antes dos indivíduos com ELA do tipo espinhal é 5,34 vezes maior.

Em relação ao sexo, os homens apresentaram um intervalo de manutenção da fala por 1.553 e 2.800 dias (4a;3m;3d e 7a;8m;5d), enquanto as mulheres apresentaram um intervalo entre 1.126 dias e 1.796 dias (3a;2m;11d e 4a;11m;6d). Observou-se que os homens mantiveram 73% da função da fala, entre 883 e 1.248 dias (2a;5m;3d e 3a;5m;3d), enquanto as mulheres mantiveram 77% desta função entre 943 e 1065 dias (2a;7m;3d e 2a;11m;5d) (Figura 1).

Em relação à perda da função da fala, o teste de Log-rank mostrou um valor p=0,567, indicando que não houve diferença estatística na progressão da perda da fala entre os dois sexos. Já a comparação de regressão de Cox determinou que o risco de indivíduos do sexo feminino perderem a função de fala antes dos do masculino é de 1,35 vezes maior.

Na análise da função de salivação, considerando o aumento da saliva acumulada na cavidade oral, observou-se que indivíduos com ELA mantiveram boa funcionalidade da salivação entre 1.553 e 1.796 dias (4a;3m;3d e 4a;11m;6d). Em um tempo estimado de 1.248 dias (3a;5m;3d), esses sujeitos ainda mantêm 65% da função de salivação, com desvio padrão de 0,12 e intervalo de confiança de 95% (Figura 2).

**Figura 2:** Curvas de Kaplan-Meier com estimativa de manutenção da função da salivação ao longo do tempo em pacientes com ELA e comparando a estimativa de manutenção de acordo com o tipo de ELA e em função do sexo dos participantes

Na Figura 2, pode-se observar a diferença na manutenção da função de salivação entre indivíduos com ELA do tipo bulbar e indivíduos com ELA do tipo espinhal. Verificou-se que os indivíduos com ELA do tipo espinhal mantêm essa função por um intervalo de tempo maior que os do tipo bulbar. Enquanto os do tipo bulbar mantêm 57% dessa função entre 304 e 518 dias (10m;4d e 1a;5m;3d), os do tipo espinhal mantêm 82% dessa função entre 1.553 e 1.796 dias (4a;3m;2d e 4a;11m;9d).

O teste de Log-rank revelou diferença estatística entre os dois tipos, com valor de p < 0,0001. A regressão de Cox demonstrou que pacientes com ELA do tipo bulbar têm risco 21,45 vezes maior de desenvolver queixa de sialorreia, em comparação aos pacientes com ELA do tipo espinhal.

Ao comparar homens e mulheres, a curva mostrou que os homens mantêm 80% da função de salivação entre 761 e 1.064 dias (2a;1m;1d e 2a;11m;4d), enquanto as mulheres mantêm 81% dessa função entre 638 e 850 dias (1a;9m;1d a 2a;4m) (Figura 2).

Quando essas variáveis foram analisadas pelo teste de Log-rank, não foi observada diferença estatística na função de salivação entre os sexos (p = 0,683). Na regressão de Cox, as mulheres apresentaram risco de evoluir para piora dessa queixa até 1,24 vezes maior que os homens.

Na análise estatística da deglutição, pelo método de Kaplan-Meier, a mediana ficou entre 0,65 e 0,48, o que indica que, para se alcançar o desfecho desse evento em indivíduos com ELA, leva-se entre 2.640 e 2.800 dias (7a;2m;25d e 7a;8m;5d), com erro-padrão de 0,12. Na Figura 3, pode-se observar que 82% dessa função é mantida entre 943 e 1.188 dias (2a;7m;3d e 3a;3m;3d).

**Figura 3:** Curvas de Kaplan-Meier com estimativa de manutenção da função da deglutição ao longo do tempo em pacientes com ELA e comparando a estimativa de manutenção de acordo com o tipo de ELA e em função do sexo dos participantes

Quando essa função foi comparada entre indivíduos com ELA do tipo bulbar e indivíduos com ELA do tipo espinhal, verificou-se que 93% da manutenção da função de deglutição está preservada entre 120 e 883 dias (4m e 2a;5m;3d) em pacientes com ELA do tipo bulbar. Já em pacientes com ELA do tipo espinhal, 90% dessa função é mantida entre 546 e 730 dias (1a;6m;3d e 2a) (Figura 3).

Nos pacientes com ELA do tipo espinhal, a função de deglutição foi mantida entre 2.800 e 3.259 dias (7a;8m;5d e 8a;11m;3d). O teste de Log-rank demonstrou que não houve diferença estatística, quando comparados indivíduos com ELA do tipo bulbar e do tipo espinhal, com valor de p=0,107.

Na regressão de Cox, o resultado indicou que indivíduos com ELA do tipo bulbar têm risco 3,24 vezes maior de perder a função de deglutição em comparação aos indivíduos do tipo espinhal.

Quando comparadas as alterações dessa função entre os sexos, o teste de Kaplan-Meier mostrou que 92% da função de deglutição é mantida em indivíduos do sexo masculino entre 761 e 1.064 dias (2a;1m;1d e 2a;11m;4d). Já em indivíduos do sexo feminino, 74% dessa função é mantida entre 943 e 1.796 dias (2a;7m;3d e 4a;11m;6d) (Figura 3).

O teste de Log-rank não demonstrou diferença nesta comparação, com valor de p=0,081. Em relação a essa variável, a regressão de Cox indicou que as mulheres têm risco 3,82 vezes maior de chegarem ao declínio mais rapidamente dessa função.

## DISCUSSÃO

Os resultados obtidos em relação à fala estão em concordância com a literatura, uma vez que as alterações dessa função tornam-se mais evidentes com a progressão da doença^(16,17)^. Os pacientes com ELA, independentemente do tipo, evoluem para a disartria e, muitas vezes, para a ausência de fala. A disartria é caracterizada pelo comprometimento das bases motoras da fala (articulação, ressonância, fonação, prosódia e respiração), normalmente associadas ao comprometimento bulbar ou do trato piramidal, alterando a velocidade, amplitude, firmeza e tornando essa função imprecisa e ininteligível, podendo evoluir para uma anartria^(18,19)^.

Quando se considera o tipo de ELA, bulbar ou espinhal, a análise demonstrou que indivíduos com ELA do tipo bulbar mantêm um percentual desta função por um período inferior aos indivíduos com ELA do tipo espinhal. Um estudo apontou que apenas 30% dos indivíduos com ELA começam com alterações bulbares^(20)^, corroborando o resultado de nosso estudo, considerando o número da amostra, que aponta um percentual maior de indivíduos com ELA do tipo espinhal. Todavia, sujeitos do tipo bulbar apresentam perda da fala em um curto período de tempo^(4)^, pois na ELA do tipo bulbar há um envolvimento do NMS, do NMI ou de ambos, localizados no córtex e tronco encefálico, o que causa dificuldade na fluência verbal e voz, tornando a fala lentificada, soprosa e hipernasalizada^(1,5,10)^.

Entretanto, tais estudos não mencionam em quanto tempo as alterações ocorrem. A análise de sobrevivência pelo método Kaplan-Meier utilizada em nosso estudo, permitiu quantificar a estimativa de manutenção da função e, consequentemente, permite tomada de decisão em relação aos momentos de intervenção terapêutica, bem como às estratégias a serem adotadas nesse processo. Esta análise evidencia a importância do uso de marcadores de tempo na clínica e pesquisa de doenças neuromusculares.

O marcador de tempo emergiu como uma ferramenta crucial na avaliação e monitoramento das doenças neuromusculares, complementando as medidas tradicionais de função muscular e qualidade de vida^(21-23)^. A importância desse marcador reside na sua capacidade de capturar mudanças sutis e progressivas ao longo do tempo, fornecendo dados sobre a progressão da doença e a eficácia das intervenções terapêuticas^(21-23)^.

Estudos anteriores^(24-26)^ demonstraram que o ritmo de progressão das doenças neuromusculares pode variar amplamente entre os indivíduos e entre diferentes tipos de doenças, como a distrofia muscular de Duchenne, a ELA e a atrofia muscular espinhal. A utilização de marcadores de tempo permite uma abordagem mais personalizada, identificando padrões de progressão que podem não ser evidentes através de avaliações clínicas esporádicas^(24-26)^.

Na ELA^(21,25)^, a avaliação longitudinal das alterações funcionais e clínico-temporais dos pacientes pode fornecer informações críticas sobre a progressão e a compreensão da variabilidade clínica da doença, bem como facilita a construção de abordagens terapêuticas personalizadas e a análise da eficácia das intervenções.

A análise pelo teste de regressão do Cox revelou que o risco de indivíduos do tipo bulbar perderem a fala antes dos sujeitos acometidos pela ELA do tipo espinhal é 5,34 vezes maior, corroborando os achados de pesquisas já realizadas em relação a essas comparações^(4,10)^.

Em relação à análise da salivação, o resultado que considerou o total dos sujeitos permitiu verificar em qual intervalo de tempo esta função é mantida. A dificuldade em expelir a saliva da cavidade oral é chamada de sialorreia, podendo causar estase e evoluir para risco de aspiração broncopulmonar, o que ocorre devido à fraqueza e incoordenação das musculaturas envolvidas na função da deglutição ocasionada pela disfagia^(27,28)^.

A aplicação da toxina botulínica costuma ser utilizada no tratamento da sialorreia em pacientes com ELA, a alteração desta função está presente em 50% destes pacientes, sugerindo que há alteração no mecanismo que envolve a deglutição de saliva^(28)^. O uso da toxina botulínica beneficia a qualidade de vida destes pacientes. A observação da progressão, como a descrita na análise deste artigo, pode indicar o momento mais propício para o uso dessa terapêutica.

Neste estudo, a evolução da perda da função de deglutição ocorreu de forma progressiva, sendo mais rápida em indivíduos com ELA do tipo bulbar do que nos do tipo espinhal. A deglutição é uma função complexa que envolve a ação neuromuscular de estruturas como o córtex, o cerebelo e o tronco encefálico, as quais estão intimamente relacionadas aos eventos orofaríngeos^(4,5,8,12)^.

De fato, nos indivíduos acometidos pela ELA do tipo bulbar, essas estruturas são primeiramente afetadas, resultando em uma degeneração que compromete rapidamente a função de deglutição. A disfagia é frequente em todas as fases da deglutição nesses indivíduos, mesmo na ausência de queixas dos pacientes, como observado em avaliações das fases preparatória oral, oral e faríngea da deglutição, com o uso da videoendoscopia da deglutição em pessoas com ELA^(29)^.

Em relação à menor manutenção da função de deglutição na ELA bulbar em comparação à ELA espinhal, outros estudos avaliaram objetivamente as manifestações orofaríngeas na ELA, comparando as formas espinhal e bulbar da doença. Esses identificaram que, em pacientes com ELA do tipo bulbar, as manifestações orofaríngeas ocorrem com mais frequência do que em pacientes com a forma espinhal^(4,8,16-18)^.

Quanto à variável sexo, em todas as análises verificadas, houve indicação de progressão mais rápida nas mulheres em relação à deglutição, à fala e à salivação. Embora outras pesquisas não façam comparação entre alterações das funções estudadas no que se refere a esta variável, um estudo sobre prevalência da ELA na cidade de Porto Alegre/RS, demonstrou que os homens são mais acometidos por esta patologia do que as mulheres^(30)^.

Ressalta-se que a análise deste estudo permitiu observar e quantificar, em dias, a manutenção da fala, da salivação e da deglutição. Esses dados são importantes para a prática clínica fonoaudiológica com pessoas com ELA, pois favorecem: o processo de avaliação dos sintomas apresentados pelos pacientes; o prognóstico; o planejamento de intervenções e a avaliação da eficácia destas; o monitoramento da progressão da doença; e a comunicação com o paciente e seus familiares

Como limitações do estudo, indica-se a exclusão de prontuários, devido, principalmente, a observação de erros ou inconsistências no preenchimento da escala ou de outros dados cadastrais. Aspectos que reforçam a importância de uma documentação adequada dos dados e a realização de treinamento com os profissionais da saúde para aplicação de escalas funcionais de forma mais eficaz. Recomenda-se a realização de estudos futuros com análises de outros tópicos da escala e com comparações entre os dados da escala funcional ao de avaliações longitudinais, clínicas e objetivas, com diferentes tipos de ELA.

## CONCLUSÃO

Há um declínio progressivo da fala, da salivação e da deglutição em pacientes com ELA ao longo de dez anos. Indivíduos com ELA do tipo espinhal mantêm essas funções por mais tempo do que aqueles com ELA do tipo bulbar. Além disso, as mulheres com ELA apresentam um risco de declínio mais rápido das funções analisadas em comparação aos homens com ELA.
